# 2,3,5-Triphenyl-2*H*-tetra­zol-3-ium bromide ethanol monosolvate

**DOI:** 10.1107/S1600536812032953

**Published:** 2012-07-28

**Authors:** Hoong-Kun Fun, Tze Shyang Chia, Gamal A. E. Mostafa, Mohamed M. Hefnawy, Hatem A. Abdel-Aziz

**Affiliations:** a X-ray Crystallography Unit, School of Physics, Universiti Sains Malaysia, 11800 USM, Penang, Malaysia; b Department of Pharmaceutical Chemistry, College of Pharmacy, King Saud University, PO Box 2457, Riyadh 11451, Saudi Arabia

## Abstract

In the title compound, C_19_H_15_N_4_^+^·Br^−^·C_2_H_5_OH, the tetra­zole ring makes dihedral angles of 57.44 (9), 50.92 (9) and 4.65 (8)° with the attached phenyl rings. In the crystal, the cation and the anion are linked to each other by inter­molecular C—H⋯Br hydrogen bonds into an infinite chain along the *b* axis. The anion and the ethanol solvent mol­ecule are linked by an O—H⋯Br hydrogen bond. The crystal studied was an inversion twin with a refined component ratio of 0.632 (5):0.368 (5).

## Related literature

For the biological activity of the triphenyl­tetra­zolium ion, see: Mostafa (2007 ▶); Hassanien *et al.* (2003 ▶); Abbas *et al.* (2001 ▶). For the stability of the temperature controller used in the data collection, see: Cosier & Glazer (1986 ▶).
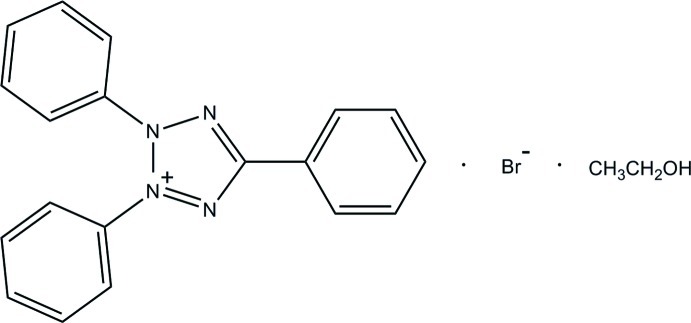


## Experimental

### 

#### Crystal data

C_19_H_15_N_4_^+^·Br^−^·C_2_H_6_O
*M* *_r_* = 425.33
Orthorhombic, 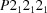

*a* = 10.0870 (11) Å
*b* = 12.1904 (13) Å
*c* = 16.5045 (18) Å
*V* = 2029.5 (4) Å^3^
*Z* = 4
Mo *K*α radiation
μ = 2.04 mm^−1^
*T* = 100 K
0.38 × 0.28 × 0.10 mm

#### Data collection

Bruker APEX DUO CCD area-detector diffractometer
Absorption correction: multi-scan (*SADABS*; Bruker, 2009 ▶) *T*_min_ = 0.514, *T*_max_ = 0.816
16773 measured reflections
5967 independent reflections
5611 reflections with *I* > 2σ(*I*)
*R*_int_ = 0.038

#### Refinement

*R*[*F* ^2^ > 2σ(*F* ^2^)] = 0.026
*wR*(*F* ^2^) = 0.064
*S* = 1.05
5967 reflections
250 parameters
H atoms treated by a mixture of independent and constrained refinement
Δρ_max_ = 0.47 e Å^−3^
Δρ_min_ = −0.45 e Å^−3^
Absolute structure: Flack (1983 ▶), 2613 Friedel pairs
Flack parameter: 0.368 (5)

### 

Data collection: *APEX2* (Bruker, 2009 ▶); cell refinement: *SAINT* (Bruker, 2009 ▶); data reduction: *SAINT*; program(s) used to solve structure: *SHELXTL* (Sheldrick, 2008 ▶); program(s) used to refine structure: *SHELXTL*; molecular graphics: *SHELXTL*; software used to prepare material for publication: *SHELXTL* and *PLATON* (Spek, 2009 ▶).

## Supplementary Material

Crystal structure: contains datablock(s) global, I. DOI: 10.1107/S1600536812032953/is5169sup1.cif

Structure factors: contains datablock(s) I. DOI: 10.1107/S1600536812032953/is5169Isup2.hkl

Supplementary material file. DOI: 10.1107/S1600536812032953/is5169Isup3.cml

Additional supplementary materials:  crystallographic information; 3D view; checkCIF report

## Figures and Tables

**Table 1:** Hydrogen-bond geometry (Å, °)

*D*—H⋯*A*	*D*—H	H⋯*A*	*D*⋯*A*	*D*—H⋯*A*
O1—H1*O*1⋯Br1	0.77 (2)	2.59 (2)	3.3434 (15)	167 (2)
C9—H9*A*⋯Br1^i^	0.93	2.83	3.7010 (18)	157
C11—H11*A*⋯Br1^ii^	0.93	2.86	3.6041 (17)	138

Symmetry codes: (i) 
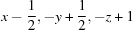
; (ii) 
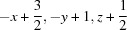
.

**Fig. 1.**
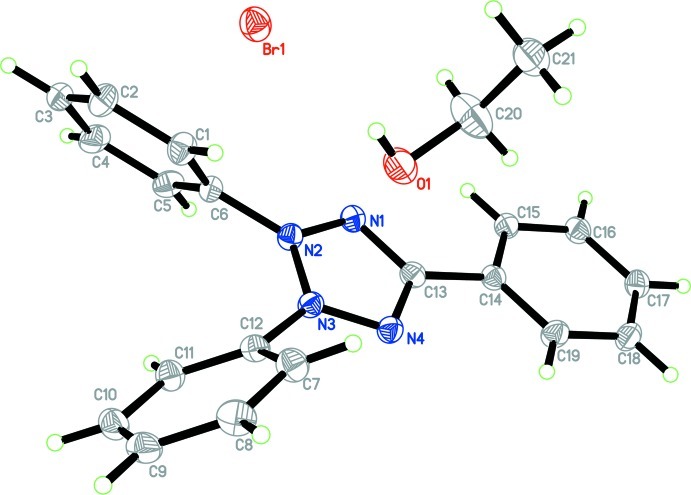
The molecular structure of the title compound with atom labels and 50% probability displacement ellipsoids.

**Fig. 2.**
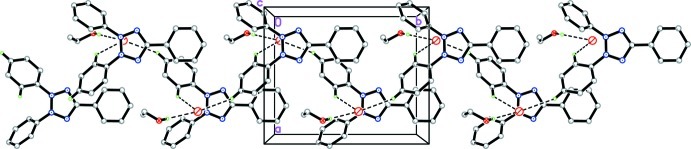
The crystal packing of the title compound. The dashed lines represent the hydrogen bonds. For clarity sake, hydrogen atoms not involved in hydrogen bonding have been omitted.

## supplementary crystallographic information

### Comment

2,3,5-Triphenyltetrazolium ion is used as indicator of bacterial dehydrogenase activity and as a reagent in colorimetric determination method for glucose dehydrogenase. Moreover, triphenyltetrazolium ion is used as ion-pair reagent for determination of antimony in waste water (Mostafa, 2007; Hassanien *et al.*, 2003; Abbas *et al.*, 2001).

The molecular structure of the title compound is shown in Fig. 1. The asymmetric unit of the title compound, C_19_H_15_N_4_^+^.Br^-^.C_2_H_6_O, consists of a 2,3,5-triphenyl-2*H*-tetrazol-3-ium cation, a bromine anion and an ethanol molecule. The three phenyl rings (C1–C6, C7–C12 and C14–C19) are twisted from the central tetrazole ring (N1–N4/C13; *r.m.s.* deviation = 0.004 Å) with dihedral angles of 57.44 (9), 50.92 (9) and 4.65 (8)°, respectively. The dihedral angles between the C1–C6 and C7–C12 rings, C1–C6 and C14–C19 rings, and C7–C12 and C14–C19 rings are 61.79 (8), 54.28 (8) and 47.03 (8)°, respectively.

In the crystal (Fig. 2), the cation and the anion are linked to each other by intermolecular C9—H9A···Br1 and C11—H11A···Br1 hydrogen bonds into an infinite chain, running along the *b* axis, with atom Br1 as the bifurcated acceptor. The crystal packing is further stabilized by the intermolecular O1—H1O1···Br1 hydrogen bond, involving the ethanol O atom.

### Experimental

Upon the addition of triphenyltetrazolium chloride solution (50 ml, 1×10^-2^*M*) to a solution of potassium bromide (50 ml), a whitish precipitate was formed. The precipitate was filtered off, washed with cold deionized water until no chloride ion was detected in the washing solution. The precipitate was dried under vacuum to give the title ion-pairs complex. Colourless blocks suitable for an X-ray structural analysis were obtained by slow evaporation from ethanol.

### Refinement

The O-bound H atom was located in a difference Fourier map and refined freely [O1—H1O1 = 0.77 (2) Å]. The remaining H atoms were positioned geometrically (C—H = 0.93, 0.96 and 0.97 Å) and refined using a riding model with *U*_iso_(H) = 1.2 or 1.5*U*_eq_(C). A rotating group model was applied to the methyl group. Seven outliers, (085), (022), (084), (043), (011), (042) and (094), were omitted in the final refinement. The crystal studied was an inversion twin with BASF of 0.368 (5).

### Crystal data

**Table:**

C_19_H_15_N_4_^+^·Br^−^·C_2_H_6_O	*F*(000) = 872
*M**_r_* = 425.33	*D*_x_ = 1.392 Mg m^−^^3^
Orthorhombic, *P*2_1_2_1_2_1_	Mo *K*α radiation, λ = 0.71073 Å
Hall symbol: P 2ac 2ab	Cell parameters from 8468 reflections
*a* = 10.0870 (11) Å	θ = 2.9–30.0°
*b* = 12.1904 (13) Å	µ = 2.04 mm^−^^1^
*c* = 16.5045 (18) Å	*T* = 100 K
*V* = 2029.5 (4) Å^3^	Block, colourless
*Z* = 4	0.38 × 0.28 × 0.10 mm

### Data collection

**Table:**

Bruker APEX DUO CCD area-detector diffractometer	5967 independent reflections
Radiation source: fine-focus sealed tube	5611 reflections with *I* > 2σ(*I*)
Graphite monochromator	*R*_int_ = 0.038
φ and ω scans	θ_max_ = 30.2°, θ_min_ = 2.4°
Absorption correction: multi-scan (*SADABS*; Bruker, 2009)	*h* = −13→14
*T*_min_ = 0.514, *T*_max_ = 0.816	*k* = −17→14
16773 measured reflections	*l* = −23→23

### Refinement

**Table:**

Refinement on *F*^2^	Secondary atom site location: difference Fourier map
Least-squares matrix: full	Hydrogen site location: inferred from neighbouring sites
*R*[*F*^2^ > 2σ(*F*^2^)] = 0.026	H atoms treated by a mixture of independent and constrained refinement
*wR*(*F*^2^) = 0.064	*w* = 1/[σ^2^(*F*_o_^2^) + (0.0382*P*)^2^] where *P* = (*F*_o_^2^ + 2*F*_c_^2^)/3
*S* = 1.05	(Δ/σ)_max_ = 0.002
5967 reflections	Δρ_max_ = 0.47 e Å^−^^3^
250 parameters	Δρ_min_ = −0.45 e Å^−^^3^
0 restraints	Absolute structure: Flack (1983), 2613 Friedel pairs
Primary atom site location: structure-invariant direct methods	Flack parameter: 0.368 (5)

### Special details

**Table:**

Experimental. The crystal was placed in the cold stream of an Oxford Cryosystems Cobra open-flow nitrogen cryostat (Cosier & Glazer, 1986) operating at 100.0 (1) K.
Geometry. All e.s.d.'s (except the e.s.d. in the dihedral angle between two l.s. planes) are estimated using the full covariance matrix. The cell e.s.d.'s are taken into account individually in the estimation of e.s.d.'s in distances, angles and torsion angles; correlations between e.s.d.'s in cell parameters are only used when they are defined by crystal symmetry. An approximate (isotropic) treatment of cell e.s.d.'s is used for estimating e.s.d.'s involving l.s. planes.
Refinement. Refinement of *F*^2^ against ALL reflections. The weighted *R*-factor *wR* and goodness of fit *S* are based on *F*^2^, conventional *R*-factors *R* are based on *F*, with *F* set to zero for negative *F*^2^. The threshold expression of *F*^2^ > σ(*F*^2^) is used only for calculating *R*-factors(gt) *etc*. and is not relevant to the choice of reflections for refinement. *R*-factors based on *F*^2^ are statistically about twice as large as those based on *F*, and *R*- factors based on ALL data will be even larger.

### Fractional atomic coordinates and isotropic or equivalent isotropic displacement parameters (Å^2^)

**Table:**

	*x*	*y*	*z*	*U*_iso_*/*U*_eq_	
Br1	0.730298 (16)	0.424198 (13)	0.338351 (9)	0.02060 (5)	
N1	0.83546 (15)	0.70430 (11)	0.62577 (8)	0.0186 (3)	
N2	0.78691 (14)	0.60438 (11)	0.62408 (8)	0.0170 (3)	
N3	0.65478 (14)	0.60760 (11)	0.62923 (8)	0.0165 (3)	
N4	0.61392 (14)	0.70935 (11)	0.63297 (9)	0.0179 (3)	
C1	0.84147 (17)	0.43874 (14)	0.54944 (10)	0.0220 (3)	
H1A	0.7743	0.4543	0.5126	0.026*	
C2	0.92042 (19)	0.34627 (14)	0.54097 (11)	0.0241 (3)	
H2A	0.9064	0.2992	0.4975	0.029*	
C3	1.01944 (18)	0.32303 (15)	0.59610 (10)	0.0236 (3)	
H3A	1.0710	0.2603	0.5899	0.028*	
C4	1.04212 (17)	0.39356 (15)	0.66111 (11)	0.0251 (3)	
H4A	1.1090	0.3778	0.6981	0.030*	
C5	0.96535 (16)	0.48744 (14)	0.67099 (10)	0.0215 (3)	
H5A	0.9799	0.5355	0.7139	0.026*	
C6	0.86633 (16)	0.50657 (13)	0.61442 (10)	0.0172 (3)	
C7	0.46152 (18)	0.51865 (15)	0.57514 (11)	0.0228 (3)	
H7A	0.4524	0.5760	0.5384	0.027*	
C8	0.37032 (17)	0.43310 (16)	0.57769 (10)	0.0257 (3)	
H8A	0.2976	0.4335	0.5430	0.031*	
C9	0.38758 (18)	0.34711 (15)	0.63185 (10)	0.0239 (3)	
H9A	0.3264	0.2900	0.6331	0.029*	
C10	0.49526 (18)	0.34559 (14)	0.68415 (10)	0.0233 (3)	
H10A	0.5065	0.2869	0.7195	0.028*	
C11	0.58660 (17)	0.43126 (15)	0.68419 (9)	0.0210 (3)	
H11A	0.6583	0.4319	0.7196	0.025*	
C12	0.56621 (16)	0.51535 (13)	0.62922 (10)	0.0177 (3)	
C13	0.72672 (18)	0.76812 (13)	0.63093 (9)	0.0177 (3)	
C14	0.72985 (18)	0.88840 (12)	0.63244 (9)	0.0172 (3)	
C15	0.85120 (17)	0.94347 (13)	0.63515 (9)	0.0189 (3)	
H15A	0.9303	0.9043	0.6357	0.023*	
C16	0.85250 (18)	1.05748 (14)	0.63703 (10)	0.0212 (3)	
H16A	0.9327	1.0950	0.6387	0.025*	
C17	0.7332 (2)	1.11546 (13)	0.63649 (10)	0.0219 (3)	
H17A	0.7344	1.1917	0.6381	0.026*	
C18	0.61291 (17)	1.06055 (15)	0.63361 (10)	0.0228 (3)	
H18A	0.5339	1.0998	0.6333	0.027*	
C19	0.61083 (17)	0.94650 (14)	0.63126 (10)	0.0213 (3)	
H19A	0.5305	0.9092	0.6289	0.026*	
O1	0.66840 (16)	0.64500 (12)	0.45240 (8)	0.0318 (3)	
C20	0.7317 (3)	0.74038 (17)	0.42248 (12)	0.0414 (5)	
H20A	0.8265	0.7327	0.4305	0.050*	
H20B	0.7024	0.8028	0.4543	0.050*	
C21	0.7067 (2)	0.76420 (17)	0.33541 (12)	0.0350 (4)	
H21A	0.7519	0.8305	0.3204	0.053*	
H21B	0.6132	0.7730	0.3267	0.053*	
H21C	0.7390	0.7045	0.3030	0.053*	
H1O1	0.680 (2)	0.6005 (18)	0.4199 (13)	0.023 (6)*	

### Atomic displacement parameters (Å^2^)

**Table:**

	*U*^11^	*U*^22^	*U*^33^	*U*^12^	*U*^13^	*U*^23^
Br1	0.01918 (7)	0.02057 (7)	0.02204 (7)	−0.00001 (6)	−0.00178 (6)	0.00324 (6)
N1	0.0194 (7)	0.0158 (6)	0.0206 (6)	−0.0008 (5)	0.0009 (5)	0.0006 (5)
N2	0.0151 (7)	0.0164 (6)	0.0195 (6)	−0.0001 (5)	−0.0012 (5)	−0.0001 (5)
N3	0.0154 (6)	0.0162 (6)	0.0180 (5)	0.0003 (5)	0.0001 (5)	−0.0004 (5)
N4	0.0177 (7)	0.0155 (6)	0.0204 (6)	0.0000 (5)	0.0009 (5)	−0.0006 (5)
C1	0.0232 (8)	0.0218 (8)	0.0210 (7)	0.0027 (6)	−0.0037 (6)	−0.0004 (6)
C2	0.0298 (9)	0.0176 (7)	0.0250 (8)	0.0004 (7)	0.0008 (7)	−0.0065 (6)
C3	0.0215 (8)	0.0195 (7)	0.0297 (8)	0.0037 (6)	0.0063 (7)	0.0038 (7)
C4	0.0193 (7)	0.0321 (9)	0.0239 (7)	0.0072 (6)	−0.0006 (7)	0.0022 (7)
C5	0.0177 (7)	0.0267 (8)	0.0201 (7)	0.0012 (6)	−0.0010 (6)	−0.0033 (7)
C6	0.0173 (7)	0.0152 (7)	0.0192 (7)	0.0017 (6)	0.0008 (6)	0.0008 (6)
C7	0.0223 (8)	0.0224 (8)	0.0236 (7)	−0.0024 (6)	−0.0013 (6)	0.0006 (6)
C8	0.0214 (8)	0.0295 (9)	0.0263 (7)	−0.0047 (8)	−0.0023 (6)	−0.0022 (8)
C9	0.0242 (8)	0.0201 (8)	0.0274 (8)	−0.0068 (6)	0.0064 (7)	−0.0058 (7)
C10	0.0284 (9)	0.0180 (7)	0.0236 (7)	−0.0032 (7)	0.0048 (7)	−0.0007 (6)
C11	0.0215 (7)	0.0195 (7)	0.0220 (7)	−0.0014 (7)	−0.0008 (5)	0.0002 (6)
C12	0.0167 (7)	0.0167 (7)	0.0198 (7)	−0.0035 (6)	0.0014 (6)	−0.0035 (6)
C13	0.0174 (6)	0.0183 (6)	0.0173 (6)	−0.0017 (7)	−0.0003 (6)	0.0007 (5)
C14	0.0183 (7)	0.0161 (6)	0.0173 (6)	−0.0008 (6)	0.0002 (6)	0.0001 (5)
C15	0.0180 (7)	0.0188 (8)	0.0200 (6)	0.0001 (6)	0.0003 (6)	0.0017 (6)
C16	0.0209 (8)	0.0183 (8)	0.0243 (7)	−0.0030 (6)	0.0006 (6)	0.0013 (6)
C17	0.0252 (8)	0.0162 (6)	0.0244 (6)	−0.0001 (7)	0.0016 (7)	0.0008 (6)
C18	0.0208 (8)	0.0188 (8)	0.0287 (7)	0.0041 (6)	0.0013 (6)	−0.0002 (7)
C19	0.0165 (8)	0.0207 (9)	0.0267 (7)	−0.0006 (6)	0.0003 (6)	−0.0003 (6)
O1	0.0459 (9)	0.0234 (6)	0.0261 (6)	0.0004 (6)	0.0077 (6)	0.0030 (5)
C20	0.0565 (14)	0.0350 (10)	0.0326 (9)	−0.0145 (11)	−0.0001 (10)	0.0063 (8)
C21	0.0472 (12)	0.0300 (9)	0.0279 (8)	0.0026 (8)	0.0066 (10)	0.0048 (8)

### Geometric parameters (Å, º)

**Table:**

N1—N2	1.3131 (18)	C10—C11	1.393 (2)
N1—C13	1.348 (2)	C10—H10A	0.9300
N2—N3	1.3360 (19)	C11—C12	1.384 (2)
N2—C6	1.445 (2)	C11—H11A	0.9300
N3—N4	1.3085 (19)	C13—C14	1.467 (2)
N3—C12	1.436 (2)	C14—C19	1.394 (2)
N4—C13	1.345 (2)	C14—C15	1.397 (2)
C1—C6	1.377 (2)	C15—C16	1.390 (2)
C1—C2	1.387 (2)	C15—H15A	0.9300
C1—H1A	0.9300	C16—C17	1.395 (3)
C2—C3	1.380 (3)	C16—H16A	0.9300
C2—H2A	0.9300	C17—C18	1.387 (3)
C3—C4	1.394 (3)	C17—H17A	0.9300
C3—H3A	0.9300	C18—C19	1.391 (2)
C4—C5	1.391 (2)	C18—H18A	0.9300
C4—H4A	0.9300	C19—H19A	0.9300
C5—C6	1.387 (2)	O1—C20	1.415 (2)
C5—H5A	0.9300	O1—H1O1	0.77 (2)
C7—C12	1.383 (2)	C20—C21	1.487 (3)
C7—C8	1.391 (2)	C20—H20A	0.9700
C7—H7A	0.9300	C20—H20B	0.9700
C8—C9	1.389 (3)	C21—H21A	0.9600
C8—H8A	0.9300	C21—H21B	0.9600
C9—C10	1.388 (3)	C21—H21C	0.9600
C9—H9A	0.9300		
			
N2—N1—C13	103.49 (14)	C12—C11—H11A	121.4
N1—N2—N3	110.09 (13)	C10—C11—H11A	121.4
N1—N2—C6	124.11 (14)	C7—C12—C11	123.91 (16)
N3—N2—C6	125.75 (14)	C7—C12—N3	116.88 (15)
N4—N3—N2	110.19 (14)	C11—C12—N3	119.16 (15)
N4—N3—C12	123.11 (14)	N4—C13—N1	112.50 (13)
N2—N3—C12	126.69 (14)	N4—C13—C14	123.39 (16)
N3—N4—C13	103.73 (14)	N1—C13—C14	124.11 (16)
C6—C1—C2	117.50 (16)	C19—C14—C15	120.73 (14)
C6—C1—H1A	121.2	C19—C14—C13	119.28 (16)
C2—C1—H1A	121.2	C15—C14—C13	119.99 (16)
C3—C2—C1	121.04 (16)	C16—C15—C14	119.30 (16)
C3—C2—H2A	119.5	C16—C15—H15A	120.3
C1—C2—H2A	119.5	C14—C15—H15A	120.3
C2—C3—C4	119.97 (16)	C15—C16—C17	119.87 (16)
C2—C3—H3A	120.0	C15—C16—H16A	120.1
C4—C3—H3A	120.0	C17—C16—H16A	120.1
C5—C4—C3	120.41 (16)	C18—C17—C16	120.68 (15)
C5—C4—H4A	119.8	C18—C17—H17A	119.7
C3—C4—H4A	119.8	C16—C17—H17A	119.7
C6—C5—C4	117.40 (16)	C17—C18—C19	119.77 (16)
C6—C5—H5A	121.3	C17—C18—H18A	120.1
C4—C5—H5A	121.3	C19—C18—H18A	120.1
C1—C6—C5	123.67 (15)	C18—C19—C14	119.63 (16)
C1—C6—N2	118.71 (15)	C18—C19—H19A	120.2
C5—C6—N2	117.62 (14)	C14—C19—H19A	120.2
C12—C7—C8	117.61 (16)	C20—O1—H1O1	105.3 (16)
C12—C7—H7A	121.2	O1—C20—C21	114.90 (19)
C8—C7—H7A	121.2	O1—C20—H20A	108.5
C9—C8—C7	120.16 (16)	C21—C20—H20A	108.5
C9—C8—H8A	119.9	O1—C20—H20B	108.5
C7—C8—H8A	119.9	C21—C20—H20B	108.5
C10—C9—C8	120.57 (16)	H20A—C20—H20B	107.5
C10—C9—H9A	119.7	C20—C21—H21A	109.5
C8—C9—H9A	119.7	C20—C21—H21B	109.5
C9—C10—C11	120.55 (16)	H21A—C21—H21B	109.5
C9—C10—H10A	119.7	C20—C21—H21C	109.5
C11—C10—H10A	119.7	H21A—C21—H21C	109.5
C12—C11—C10	117.17 (15)	H21B—C21—H21C	109.5
			
C13—N1—N2—N3	−0.90 (17)	C8—C7—C12—C11	1.3 (3)
C13—N1—N2—C6	176.61 (14)	C8—C7—C12—N3	−176.24 (15)
N1—N2—N3—N4	1.10 (19)	C10—C11—C12—C7	0.0 (3)
C6—N2—N3—N4	−176.36 (13)	C10—C11—C12—N3	177.52 (15)
N1—N2—N3—C12	179.91 (13)	N4—N3—C12—C7	49.6 (2)
C6—N2—N3—C12	2.5 (2)	N2—N3—C12—C7	−129.11 (18)
N2—N3—N4—C13	−0.76 (17)	N4—N3—C12—C11	−128.14 (17)
C12—N3—N4—C13	−179.62 (14)	N2—N3—C12—C11	53.2 (2)
C6—C1—C2—C3	0.5 (3)	N3—N4—C13—N1	0.20 (17)
C1—C2—C3—C4	−0.6 (3)	N3—N4—C13—C14	178.98 (14)
C2—C3—C4—C5	0.1 (3)	N2—N1—C13—N4	0.43 (17)
C3—C4—C5—C6	0.5 (3)	N2—N1—C13—C14	−178.33 (13)
C2—C1—C6—C5	0.2 (3)	N4—C13—C14—C19	−3.8 (2)
C2—C1—C6—N2	179.09 (15)	N1—C13—C14—C19	174.82 (15)
C4—C5—C6—C1	−0.7 (3)	N4—C13—C14—C15	176.09 (14)
C4—C5—C6—N2	−179.57 (15)	N1—C13—C14—C15	−5.3 (2)
N1—N2—C6—C1	−120.81 (18)	C19—C14—C15—C16	0.3 (2)
N3—N2—C6—C1	56.3 (2)	C13—C14—C15—C16	−179.62 (15)
N1—N2—C6—C5	58.2 (2)	C14—C15—C16—C17	0.2 (2)
N3—N2—C6—C5	−124.72 (17)	C15—C16—C17—C18	−0.4 (2)
C12—C7—C8—C9	−1.4 (3)	C16—C17—C18—C19	0.0 (2)
C7—C8—C9—C10	0.3 (3)	C17—C18—C19—C14	0.5 (3)
C8—C9—C10—C11	1.1 (3)	C15—C14—C19—C18	−0.6 (2)
C9—C10—C11—C12	−1.2 (2)	C13—C14—C19—C18	179.27 (15)

### Hydrogen-bond geometry (Å, º)

**Table:**

*D*—H···*A*	*D*—H	H···*A*	*D*···*A*	*D*—H···*A*
O1—H1*O*1···Br1	0.77 (2)	2.59 (2)	3.3434 (15)	167 (2)
C9—H9*A*···Br1^i^	0.93	2.83	3.7010 (18)	157
C11—H11*A*···Br1^ii^	0.93	2.86	3.6041 (17)	138

Symmetry codes: (i) *x*−1/2, −*y*+1/2, −*z*+1; (ii) −*x*+3/2, −*y*+1, *z*+1/2.
